# Chicken-Specific Kinome Analysis of Early Host Immune Signaling Pathways in the Cecum of Newly Hatched Chickens Infected With *Salmonella enterica* Serovar Enteritidis

**DOI:** 10.3389/fcimb.2022.899395

**Published:** 2022-06-30

**Authors:** Michael H. Kogut, Kenneth J. Genovese, J. Allen Byrd, Christina L. Swaggerty, Haiqi He, Yuhua Farnell, Ryan J. Arsenault

**Affiliations:** ^1^ Southern Plains Agricultural Research Center, United States Department of Agriculture-Agricultural Research Service (USDA ARS), College Station, TX, United States; ^2^ Department of Poultry Science, Texas A&M University, College Station, TX, United States; ^3^ Department of Animal and Food Sciences, University of Delaware, Newark, DE, United States

**Keywords:** *Salmonella*, chicken, kinome, phosphorylation, innate immunity, immune phenotype

## Abstract

Poultry is a major source of human foodborne illness caused by broad host range *Salmonella* serovars (paratyphoid), and developing cost-effective, pre-harvest interventions to reduce these pathogens would be valuable to the industry and consumer. Host responses to infectious agents are often regulated through phosphorylation. However, proteomic mechanisms of Salmonella acute infection biology and host responses to the bacteria have been limited concentrating predominately on the genomic responses of the host to infection. Our recent development of chicken-specific peptide arrays for kinome analysis of host phosphorylation-based cellular signaling responses provided us with the opportunity to develop a more detailed understanding of the early (4-24 h post-infection) host-pathogen interactions during the initial colonization of the cecum by Salmonella. Using the chicken-specific kinomic immune peptide array, biological pathway analysis showed infection with *S*. Enteritidis increased signaling related to the innate immune response, relative to the non-infected control ceca. Notably, the acute innate immune signaling pathways were characterized by increased peptide phosphorylation (activation) of the Toll-like receptor and NOD-like receptor signaling pathways, the activation of the chemokine signaling pathway, and the activation of the apoptosis signaling pathways. In addition, *Salmonella* infection induced a dramatic alteration in the phosphorylation events of the JAK-STAT signaling pathway. Lastly, there is also significant activation of the T cell receptor signaling pathway demonstrating the initiation of the acquired immune response to *Salmonella* infection. Based on the individual phosphorylation events altered by the early *Salmonella* infection of the cecum, certain conclusions can be drawn: (1) *Salmonella* was recognized by both TLR and NOD receptors that initiated the innate immune response; (2) activation of the PPRs induced the production of chemokines CXCLi2 (IL-8) and cytokines IL-2, IL-6, IFN-α, and IFN-γ; (3) *Salmonella* infection targeted the JAK-STAT pathway as a means of evading the host response by targeting the dephosphorylation of JAK1 and TYK2 and STAT1,2,3,4, and 6; (4) apoptosis appears to be a host defense mechanism where the infection with Salmonella induced both the intrinsic and extrinsic apoptotic pathways; and (5) the T cell receptor signaling pathway activates the AP-1 and NF-κB transcription factor cascades, but not NFAT.

## Introduction

*Salmonella* invasion of the chicken cecum induces a short-lived inflammation mediated by the increased expression of pro-inflammatory cytokine and chemokine genes in the intestinal tissue (Withanage et al., 2005; Setta et al., 2012; Matulova et al., 2013; Rychlik et al., 2014). The activation of the innate immune response induces an influx of heterophils (granulocytes) to the intestine that limits bacterial invasion (Kogut et al., 1994; Kogut et al., 2012) but does not lead to a pathological inflammation that is seen in mammals (Foster et al., 2003; Patel and McCormick, 2014). However, this heterophil infiltration of the intestine does not have a significant protective response against the salmonellae bacteria that remain in the lumenal side of the ceca. Interestingly, this inflammatory response is largely resolved by 2-3 days post-infection (Kogut et al., 2012; Kogut et al., 2016; Kogut et al., 2017) and is characterized by the reduction of pro-inflammatory cytokines mRNA transcription in the cecum to non-infected control levels yet *Salmonella* can persist in the intestine and be shed in the feces for several weeks (Withanage et al., 2005).

We have previously used a chicken-specific kinome peptide array analysis to define changes in cecal tissue phenotype during the establishment of a *Salmonella* persistent infection in chickens. Using the kinome array, we described a *Salmonella*-mediated reprogramming of host signaling pathways around 3 days post-infection to be the principal mechanism developed by the bacteria to establish a successful persistent long-term colonization in chicken cecum (Kogut et al., 2017). A wide range of host immune and metabolic pathways acted as *Salmonella* targets, including the T cell receptor signaling pathway, JAK-STAT signaling pathway, the Wnt-Ca^2+^ signaling pathway, NF-κB signaling, the mTOR and AMPK metabolic signaling pathways (Kogut and Arsenault, 2015; Kogut et al., 2017; Kogut and Arsenault, 2017). These pathways control cellular functions such as immunity and metabolism and are an indication of the complexity and scale with which *Salmonella* infection interferes and thwarts host defense mechanisms to establish and maintain long-term cecal colonization.

However, until now, there has been no characterization of the acute host signaling interactome in the chicken intestine during the initial (first 24 h) infection with *Salmonella*. In the murine *Salmonella* infection model, *Salmonella enterica* serovars use self-induced pathologic inflammation which provides a growth advantage in the host gut that favors intestinal colonization (Winter et al., 2010; Winter and Baumler, 2011; Thiennimitr et al., 2012; Patel and McCormick, 2014). An inflammatory response is generated in chickens, but the signaling pathways that are activated or altered are unknown. In this study, we used a chicken-specific global immunologic peptide array to study the changes in cecal immune signaling pathways during the first 24 h following *Salmonella* Enteritidis infection.

## Materials and Methods

### Ethics Statement

These studies were approved by the Animal Care and Use Committee (ACUC) at the Southern Plains Agricultural Research Center, Agricultural Research Service, United States Department of Agriculture (ACUC #2012007), which meets all federal requirements as defined in the Animal Welfare Act, the Public Health Service Policy, and the Humane Care and Use of Laboratory Animals.

### Experimental Animals

Experiments were conducted according to the regulations established by the United States Department of Agriculture Animal Care and Use Committee. Broiler chickens used in this study were obtained from a commercial breeder and were all the same genetic background and were not vaccinated at any time. Chicks were placed in floor pens containing wood shavings, provided supplemental heat, water, and a balanced, unmedicated corn and soybean meal-based chick starter diet ad libitum that met or exceeded the levels of critical nutrients recommended by the National Research Council (1994). *Salmonella* was not detected in the feed or from the paper tray liners.

### *S. Enteritidis* Challenge

A poultry isolate of *Salmonella enterica* serovar Enteritidis (S. Enteritidis; (ID 9711771, part 24) was obtained from the National Veterinary Services Laboratory (Ames, IA, USA), and was selected for resistance to nalidixic acid and novobiocin and maintained in tryptic soy broth (Difco Laboratories, Sparks, MD, USA) containing antibiotics (20 μg/mL nalidixic acid and 25 μg/mL novobiocin; Sigma Chemical Co.; St. Louis, MO, USA). A stock culture was prepared in sterile PBS and adjusted to a concentration of 1 x 10^9^ colony forming units (CFU/mL). The viable cell concentration of the challenge dose for each experiment was determined by colony counts on XLT4 agar base plates with XLT4 supplement (Difco) and nalidixic acid and novobiocin (XLT-NN).

### Experimental Design

One-day-old broiler chickens were randomly distributed into two experimental groups: non-infected control and infected. Each group contained 15 birds fed a balanced, unmedicated corn and soybean meal-based diet. Two days post-hatch, all chickens were orally challenged with either 1 mL of 5 x 10^6^ CFU/mL S. Enteritidis (infected group) or mock challenged with 1 mL sterile PBS (non-infected group). At 4, 8 and 24 hours after challenge, 4 chickens from each group were humanly euthanized; all euthanasia procedures followed the guidelines set down in the American Veterinary Medical Association (AVMA) Guidelines for the Euthanasia of Animals. Cecal contents were analyzed for S. Enteritidis colonization and cecal tissue was flash frozen in liquid nitrogen and stored at -80°C for use in the peptide arrays. The experiment was conducted three times. Therefore, the ceca from a total of 15 chickens for each of the 2 groups (5 chickens each in 3 experiments) were used.

### Sample Collection to Confirm Bacteria Presence

The ceca from each chicken were removed aseptically, and the contents (0.25 g) were added to tetrathionate broth for enrichment, as well as serially diluted to 1:10, 1:100, 1:1,000, or 1:10,000, and spread onto selection plates. Diluent was produced by adding tissue to 1 mL Butterfield’s Buffered Phosphate Diluent [Sigma Aldrich (St. Louis, MO)]. Selection antibiotics for SE included 20 μg/mL naladixic acid and 25 μg/mL novobiocin. The plates and enrichment tubes were incubated at 37°C for 24 h. The presence of resistant SE per gram of cecal contents was determined by colony counts on the plates. Contents of enrichment tubes were plated and incubated for an additional 24 h.

### Statistical Analysis of SE Cecal Colonization

Statistical differences between groups were determined by analysis of variance (P ≤ 0.05). Means were further separated for significance with a pair-wise multiple comparison procedure (Tukey test, P ≤ 0.05). Chi square analysis was used to determine significant differences between groups in *Salmonella* cecal colonization rates. Differences were significant based on the 0.05 level of probability. The enrichment data were expressed as positive/total chickens (%) and the percent recovery of *S*. Enteritidis was compared using the chi-squared test of independence to determine the significance (P ≤ 0.05) for these studies.

### Chicken-Specific Kinome (Peptide) Array

For the phenotype readout, a peptide array was used to provide tissue immunometabolism information from the host. At 3 time points (4, 8, and 24 h), 3 whole ceca from 3 randomly selected birds were defrosted for analysis (27 samples total for all 3 d). Each sample was weighed to obtain a consistent 40-mg sample for the array. The samples were homogenized with the Omni International Bead Ruptor Elite (Kennesaw, GA) in 100 mL of lysis buffer (20 mmol Tris-HCl pH 7.5, 150 mmol NaCl, 1 mmol EDTA, 1 mmol ethylene glycol tetraacetic acid, 1% Triton X-100, 2.5 mmol sodium pyrophosphate, 1 mmol Na3VO4, 1 mmol NaF, 1 mg/mL leupeptin, 1 g/mL aprotinin, and 1 mmol phenylmethylsulfonyl fluoride). All products were obtained from Sigma Aldrich (St. Louis, MO), unless indicated. After homogenization, the peptide array protocol was carried out according to Jalal et al. (2009) with alterations described in the study by Arsenault et al. (2017). The resulting tissue lysates were applied onto the PepStar peptide microarrays customized by JPT Peptide Technologies GmbH (Berlin, Germany) and incubated in a sealed container placed in a 5% CO_2_ incubator at 37°C for 2 h. After incubation, sample residues were washed off the arrays and the arrays were stained in phospho-specific fluorescent ProQ Diamond Phosphoprotein Stain (Life Technologies, Carlsbad, CA, USA) for 1 h. The arrays were submerged in a destain solution containing 20% acetonitrile (EMD Millipore Chemicals, Billerica, MA, USA) and 50 mM sodium acetate (Sigma-Aldrich, St. Louis, MO, USA) to remove non-phospho-specific binding. The arrays were scanned in a Tecan PowerScanner microarray scanner (Tecan Systems, San Jose, CA, USA) at 532 to 560 nm with a 580-nm filter to detect dye fluorescence. The images of the scanned array were gridded manually to fit the phospho-specific spots and extract signal intensity using GenePix Pro software (version 7.2.29 1, Molecular Devices, CA, USA). Microsoft Excel 2016 (Redmond, WA).

### Data Analysis: Kinome Array

Data normalization was performed for the kinome array, based on the study by Li et al. (2012) using the PIIKA2 online platform (http://saphire.usask.ca/saphire/piika/index.html), a tool designed for *in silico* analysis of phosphorylation sites (Trost et al., 2013). The array data were analyzed by conducting variance stabilization normalization and then performing t test, clustering and pathway analysis for statistical data. Gene ontology and Kyoto Encyclopedia of Genes and Genomes (KEGG) pathway analysis were performed by uploading the statistically significant peptide lists to the Search Tool for the Retrieval of Interacting Genes (STRING) (Szklarczyk et al., 2017).

## Results

### Salmonella Infection

At 4-, 8-, and 24-hours post-infection, animals were sacrificed, and samples collected. Infection status was confirmed by *Salmonella* Enteritidis culturing of cecal contents and feces from each animal with and without enrichment. Cultures confirmed that the infected group displayed *Salmonella* infection throughout the 24 h experiment, while *Salmonella* was not isolated from animals in the control group (**Table 1**).

**Table 1:** Number of *Salmonella* organisms in cecal contents of chickens after oral administration on day 1 of age^a^.

Mean log10 CFU of *Salmonella enterica* serovar Enteritidis of tissue (SEM) at h post-inection		
**4 h**	**8 h**	**24 h**
4.71 (0.41)	4.77 (0.33)	6.12 (0.51)

a Oral infection with 1 x 10^5^ CFU/bird Salmonella enterica serovar Enteritidis ay day-of-age broiler chickens. Viable counts are mean values from five birds at each time point.

### Kinome Arrays

Kinome analysis was carried out on the cecal samples from non-infected and infected chickens. The results from three animals from each group (*S*. Enteritidis-infected and non-infected) and time point were combined to provide a representative result. To remove any non-specific or baseline phosphorylation signal from the analysis data from each time point was corrected using the matched uninfected controls. The kinome data were subjected to pathway overrepresentation analysis to determine which cellular pathways/processes are altered under the infected and non-infected conditions. To ensure that the identified pathways represent conserved and consistent biological responses, input data were limited to peptides with a consistent pattern of differential phosphorylation across the three biological replicates in at least one of the treatment sets as well as significant changes (p ≤ 0.05) in phosphorylation level relative to the non-infected control treatment. These select data from the three animals were merged to generate a representative data set for each treatment condition. All peptides that showed significant phosphorylation changes relative to control (p ≤ 0.05) for each time point were input into the Search Tool for the Retrieval of Interacting Genes/Proteins (STRING) database (Szklarczyk et al., 2017). Using STRING functionality, KEGG pathway results were generated for each dataset.

### Biological Process Analysis

We next considered the individual peptide phosphorylation results generated by the PIIKA2 online analysis platform (Trost et al., 2013). These data are a series of fold changes and significance values for each peptide on the array from each sample analyzed. These values are generated by comparing treatment/challenge tissue array outputs to non-challenged control array outputs. The STRING protein-protein interaction database (Szklarczyk et al., 2017) generates GO Biological Process terms and a false discovery rate (FDR) which indicates the likelihood of a biological process being truly represented in the data and not generated by random chance. The top 10 biological processes for each group were compared to find the differences between the lists generated for *Salmonella*-challenged group and the non-infected control group (**Table 2**). The results provided convincing evidence for the significant involvement of the local cecal immune response, specifically the innate response, during the first 24 h post-infection with *S.* Enteritidis.

**Table 2:** Top 10 GO Biological processes altered between the *Salmonella*-infected and noninfected groups at 4, 8 and 24 h post-infection from STRING analysis.

		4 hours		8 hours		24 hours	
GO ID	Term	# peptides	*p*-value (FDR)	# peptides	*p*-value (FDR)	# peptides	*p*-value (FDR)
00450857	Innate immune response	91	1.94 x 10^-76^	80	2.20 x 10^-66^	74	1.32 x 10^-62^
0007169	Transmembrane receptor protein tyrosine kinase signaling pathway	77	7.12 x 10^-69^	65	2.83 x 10^-56^	67	7.07 x 10^-63^
0007167	Enzyme linked receptor protein signaling pathway	81	1.77 x 10^-65^	69	4.89 x 10^-54^	73	3.05 x 10^-63^
0050776	Regulation of immune response	78	3.06 x 10^-64^	65	2.96 x 10^-50^	64	3.96 x 10^-53^
0038093	Fc receptor signaling pathway	55	1.69 x 10^-63^	51	2.25 x 10^-59^	48	6.6 x 10^-57^
0002764	Immune response-regulating signaling pathway	65	2.21 x 10^-60^	56	6.73 x 10^-51^	55	3.07 x 10^-52^
0006955	Immune response	87	5.80 x 10^-60^	79	3.97 x 10^-55^	72	1.25 x 10^-50^
0006952	Defense response	86	6.13 x 10^-58^	76	6.73 x 10^-51^	65	1.77 x 10^-41^
0038095	Fc-epsilon receptor signaling pathway	47	1.79 x 10^-56^	44	1.19 x 10^-53^	42	3.07 x 10^-52^
0002768	Immune response-regulating cell surface receptor signaling pathway	58	1.76 x 10^-55^	52	9.28 x 10^-50^	50	1.85 x 10^-43^

Peptides that displayed a statistically significant change (FDR corrected) in phosphorylation state were input into the STRING database for each time point. GO Biological Process results for each time point are listed, excluding generic results that are inherent to the analysis such as “protein phosphorylation”, “enzyme linked receptor protein signaling pathway” and “intracellular signaling transduction”. These provide no relevant data for analysis.

The STRING generated KEGG pathway results showed several pathways altered by the *S*. Enteritidis infection at a statistically significant level (p ≤ 0.05 false discovery rate (FDR) corrected). Of particular interest were the immune pathways that contained substantial numbers of peptides that were significantly differentially phosphorylated at multiple times over the course of the study. A subset of these pathways is shown in **Table 3**. Specifically, there were a total of 139 different significantly altered phosphorylation events within these 6 immune signaling pathways at the 4-hour post-infection time point, 120 at the 8-hour time point and another 124 after 24 h post-infection.

**Table 3:** Kegg pathways generated by Search Tool for the Retrieval of Interacting Genes/Proteins (STRING) with consistent altered phosphorylation across all time points.

		4 hours pi		8 hours pi		24 hours pi	
GO ID	Pathway	# peptides	*p*-value (FDR)	# peptides	*p*-value (FDR)	# peptides	*p*-value (FDR)
**hsa04660**	T cell receptor signaling pathway	30	8.74 x 10^-24^	31	8.82 x 10^-27^	26	3.84 x 10^-21^
**hsa04062**	Chemokine signaling pathway	35	4.86 x 10^-20^	28	8.46 x 10^-15^	27	1.34 x 10^-14^
**has04620**	Toll-like receptor signaling pathway	23	4.55 x 10^-15^	18	7.98 x 10^-11^	24	8.76 x 10^-18^
**hsa04630**	JAK-STAT signaling pathway	25	1 x 10^-13^	19	1.85 x 10^-9^	20	7.18 x 10^-11^
**hsa04621**	NOD receptor signaling pathway	12	8.65 x 10^-8^	8	6.39 x 10^-4^	12	1.23 x 10^-8^
**hsa04210**	Apoptosis	14	1.85 x 10^-7^	16	1.38 x 10^-9^	15	2.01 x 10^-9^

Peptides that displayed a statistically significant change in phosphorylation state were input into the STRING database for each time point. Generated pathways that displayed a p-value of less than 0.05 (FDR corrected) are listed.

### Phosphorylation Events Within Specific Pathways

#### Toll-Like Receptor Pathway

Toll-like receptors (TLRs) initiate a rapid activation of innate immunity by inducing production of pro-inflammatory cytokines and chemokines. As shown in **Table 4** and **Supplemental Figure 1**, TLR signaling pathways in the cecum phosphorylated by *S*. Enteritidis infection appears to be MyD88-dependent with: (1) the phosphorylation (activation) of NF-κB and MAPK signaling pathways and (2) associated with the phosphorylation of and IRF7 induction of interferon-α/β and the activation of the JAK-STAT pathway through the phosphorylation of IFNAR1 receptor.

**Table 4:** Members of the TLR signaling pathway showing differential phosphorylation between *Salmonella enterica* serovar Enteritidis-infected cecal tissue and non-infected cecal tissue.

			4 hours pi		8 hours pi		24 hours pi	
Peptide name	UniProt ID	p-site*	Fold change	*p*-value	Fold change	*p*-value	Fold change	*p*-value
**AKT1**	P31749	S473	1.245	0.000	1.126	0.030	1.361	0.00002
**AKT3**	Q94243	S472	1,278	0.00006	1.175	0.016	–	–
**PIK3R1**	P27986	S608	1.907	0.000	1.107	0.014	–	–
**PIK3R2**	O00459	Y612	1.670	0.006	1.521	0.036	–	–
**IFNAR1**	P17181	S547	1.408	0.000	1.305	0.000	1.155	0.003
**TIRAP**	P58753	Y179	1.119	0.027	1.205	0.000	–	–
**FOS**	P01100	S349	1.199	0.004	–	–	1.187	0.005
**CHUK**	Q015111	S194	1.086	0.017	–	–	1.262	0.002
**IRF7**	Q92985	S464	1.393	0.00002	–	–	1.223	0.009
**CASP8**	Q14790	S347	1.335	0.0003	1.095	0.36	1.305	0.001
**NFκB-p105**	P19838	S342	1.803	0.00001	1.233	0.004	–	–
**MEK4**	P45984	S49	1.322	0.001	–	–	–	–
**p38**	Q16539	Y323	1.314	0.005	1.136	0.038	1.228	0.03
**TAK1**	O43318	S446	1.16	0.006	–	–	–	–
**MEK1**	Q02750	S300	1.132	0.036	1.231	0.0003	1.105	0.02
**ERK1**	P27361	T193	1.16	0.029	1.194	0.00001	–	–
**JNK1**	P45983	Y185	1.41	0.927	ND	0.193	1.201	0.023
**TRAF2**	Q12933	T117	1.091	0.027	1.178	0.0003	1.091	0.031
**RAC1**	P63000	S71	1.242	0.0004	1.23	0.0004	1.314	0.00001

Selection of peptides differentially phosphorylated between non-infected group and S. Enteritidis challenge group in the cecum. The UniProt ID identifies the protein, while the p-site identifies the specific phosphorylation target site on that protein. Fold-Change indicates the directionality of phosphorylation status for each treatment compared to control. The p-value is the measure of significance (α = 0.05). UniProt IDs and p-site correspond to human proteins for annotation purposes. ND = no statistical difference in phosphorylation between S. Enteritidis-infected and non-infected tissue.

*= Phosphorylation site.

#### NOD-Like Receptor Pathway

Cecal infection by *S.* Enteritidis induced the activation of the NOD-like receptor (NLR) signaling pathway within 4-hours post-infection probably *via* the prototypic NOD-1 pathway which sense the cytosolic presence of bacterial peptidoglycan fragments that escaped from endosomal compartments, driving the activation of both the NF-κB and MAPK signaling pathways. Based on the phosphorylation events shown in **Table 5** and **Supplemental Figure 2**, the NOD-1 pathway is characterized by the phosphorylation of RIP2, CARD9, TRAF2, TAK1, TAB that leads to the activation of IκB that activates the NF-κB pathway that leads to pro-inflammatory cytokine and chemokine production and the phosphorylation (activation) of the MAPK (ERK1/3, JNK1, and p38 proteins) and AP-1 (FOS and JUN) signaling pathways that results in pro-inflammatory cytokine and chemokine production (**Table 5**; **Supplemental Figure 3**).

**Table 5:** Members of the NOD-like receptor signaling pathway showing differential phosphorylation between *Salmonella enterica* serovar Enteritidis-infected cecal tissue and non-infected cecal tissue.

			4 hours pi		8 hours pi		24 hours pi	
Peptide name	UniProt ID	p-site*	Fold change	*p*-value	Fold change	*p*-value	Fold change	*p*-value
**NFκB-p105**	P19838	S342	1.803	0.00001	1.233	0.004	–	–
**p38**	Q16539	Y323	1.314	0.005	1.136	0.038	1.228	0.03
**TAK1**	O43318	S446	1.16	0.006	–	–	1.215	0.03
**ERK1**	P27361	T193	1.16	0.029	1.194	0.00001	–	–
**JNK1**	P45983	Y185	1.41	0.927	–	–	1.201	0.023
**FOS**	P01100	S349	1.199	0.004	–	–	1.187	0.005
**JUN**	P05412	S69	1.138	0.032	1.178	0.033	–	–
**CHUK**	Q015111	S194	1.086	0.017	–	–	1.262	0.002
**RIPK2**	Q13546	Y384	1.170	0.047	1.151	0.001	–	–
**XIAP**	P98170	S87	-1.752	0.00001	–	–	-1.312	0.010
**TRAF2**	Q12933	T117	1.091	0.027	1.178	0.0003	1.091	0.031
**CARD9**	Q9H257	S238	1.227	0.006	–	–	1.217	0.001
**TAB3**	Q8N5C8	S60	1.134	0.037	1.214	0.003	1.097	0.032
**ERK3**	Q16659	S189	1.314	0.0002	1.212	0.000	–	–
**IkB**	P25963	Y46	1.193	0.038	1.178	0.009	–	–

Selection of peptides differentially phosphorylated between non-infected group and S. Enteritidis challenge group in the cecum. The UniProt ID identifies the protein, while the p-site identifies the specific phosphorylation target site on that protein. Fold-Change indicates the directionality of phosphorylation status for each treatment compared to control. The p-value is the measure of significance (α = 0.05). UniProt IDs and p-site correspond to human proteins for annotation purposes.

*= Phosphorylation site.

#### Chemokine Signaling Pathways

Following infection with *S*. Enteritidis, a number of chemokine signaling-associated pathways were found to be altered when compared to the non-infected control tissues: MAPK-JAK-STAT signaling pathways (**Table 6A**), the PI3K signaling pathway (**Table 6B**), and the CCR5P-PLC-PKC signaling pathway (**Table 6C**) (**Supplemental Figure 3**). The most consistent observation in all three downstream pathways regardless of ultimate functional activity is that there was a change in the phosphorylation state of a significant number of proteins within the pathway induced within 4 hours of infection, maintained through 8 hours post-infection, but essentially returned to control levels by 24 hours post-infection. These data provide evidence that the NF-κB-mediated activation of pro-inflammatory functions induced by the MAPK-JAK-STAT signaling pathways (**Table 6A**), the actin cytoskeleton regulation and leukocyte migration activity mediated by the PI3K signaling pathway (**Table 6B**), and the migration, NO induction, and ROS production mediated by the CCR5P-PLC-PKC signaling pathway (**Table 6C**) are rapidly induced by the host in response to the bacterial colonization. Chemokine-mediated signaling leading to cytoskeletal rearrangements allow cell polarization towards the chemokine gradient that will lead to acquisition of a migratory phenotype (Wang et al., 2013).

**Table 6A:** Members of the chemokine signaling pathway showing differential phosphorylation between *Salmonella enterica* serovar Enteritidis-infected cecal tissue and non-infected cecal tissue: MAPK and JAK-STAT signaling pathways.

				4 hours pi		8 hours pi		24 hours pi	
Functions	Peptide name	UniProt ID	p-site*	Fold change	*p*-value	Fold change	*p*-value	Fold change	*p*-value
**Cytokine production** **Cellular growth & development** **Cell survival** **Apoptosis** **Cell migration**	Src	P12931	S17	2.136	0	1.268	0.02	–	–
	Shc1	P29353	Y47	1.36	0	-1.249	0.01	–	–
	GRB2	P62993	Y209	–	–	1.129	0.001	–	–
	SOS1	Q07889	S1208	1.708	0.004	1.42	0.006	1.992	0.042
	H-RAS	P01112	T35	1.695	0.001	1.26	0	–	–
	Raf-1	P10398	S343	1.219	0.003	1.287	0.032	–	–
	MEK1	Q02750	S300	1.133	0.037	1.231	0.0003	1.105	0.02
	ERK1	P27361	T193	1.160	0.029	1.194	0	–	–
	CHUK (IκκA)	O15111	S194	1.086	0.017	–	–	1.262	0.002
	NFκB1A (IκB)	P25963	1.193 - 1.086	0.04 - 0.02	1.178 - -	0.01 - -	- - 1.262	- - 0.003	1.193 - 1.086
	NFκB-p105	P19838	S342	1.803	0.00001	1.233	0.004	–	–
	NFκB-p100	QOO653	S872	1.926	0	1.214	0.02	–	–
	JAK2	O60674	Y219	1.118	0.006	1.512	0.000	1.073	0.05
	JAK3	P52333	Y980	1.135	0.032	1.185	0.017	1.217	0.0004
	STAT5B	P51672	Y740	2.208	0	1.073	0.029	1.514	0.006

Selection of peptides differentially phosphorylated between non-infected group and S. Enteritidis challenge group in the cecum. The UniProt ID identifies the protein, while the p-site identifies the specific phosphorylation target site on that protein. Fold-Change indicates the directionality of phosphorylation status for each treatment compared to control. The p-value is the measure of significance (α = 0.05). UniProt IDs and p-site correspond to human proteins for annotation purposes.

*= Phosphorylation site.

**Table 6B:** Members of the chemokine signaling pathway showing differential phosphorylation between *Salmonella enterica* serovar Enteritidis-infected cecal tissue and non-infected cecal tissue: PI3K signaling pathway.

				4 hours pi		8 hours pi		24 hours pi	
Functions	Peptide name	UniProt ID	p-site*	Fold change	*p*-value	Fold change	*p*-value	Fold change	*p*-value
**Cytokine production** **Leukocyte migration** **Chemotaxis** **Actin cytoskeleton regulation** **Cellular shape change**	PIK3R1	P27986	S608	1.907	0	1.107	0.014	–	–
	PIK3R2	O00459	Y612	1.670	0.006	1.521	0.036	–	–
	AKT1	P31749	S473	1.245	0.03	1.126	0.03	1.361	0
	CHUK (IκκA)	O15111	S194	1.086	0.017	–	–	1.262	0.002
	NFκB1A (IκB)	P25963	1.193 - 1.086	0.04 - 0.02	1.178 - -	0.01 - -	- - 1.262	- - 0.003	1.193 - 1.086
	NFκB-p105	P19838	S342	1.803	0.00001	1.233	0.004	–	–
	NFκB-p100	QOO653	S872	1.926	0	1.214	0.02	–	–
	ITK	Q08881	Y523	2.136	0	1.268	0.2	1.194	0.016
	CDC42	P60953	Y64	1.418	0	1.218	0.005	–	–
	WASP	P42786	Y256	1.484	0	1.272	0.04	–	–
	RhoA	P61586	S188	–	–	–	–	-1.174	0
	ROCK2	O75116	Y500	1.231	0.002	-1.266	0	-1.168	0.01
	FAK	Q05397	Y397	1.220	0.001	-1.263	0	-1.143	0.001
	CRK	P46108	Y252	1.806	0.001	–	–	-1.402	0
	PYK2	Q14289	Y405	1.259	0.001	–	–	-1.402	0
	PAK1	Q13153	T212	1.442	0.019	1.275	0.04	–	–
	RAC1	P63000	S71	1.252	0	1.23	0	1.314	0

Selection of peptides differentially phosphorylated between non-infected group and S. Enteritidis challenge group in the cecum. The UniProt ID identifies the protein, while the p-site identifies the specific phosphorylation target site on that protein. Fold-Change indicates the directionality of phosphorylation status for each treatment compared to control. The p-value is the measure of significance (α = 0.05). UniProt IDs and p-site correspond to human proteins for annotation purposes.

*= Phosphorylation site.

**Table 6C:** Members of the chemokine signaling pathway showing differential phosphorylation between *Salmonella enterica* serovar Enteritidis-infected cecal tissue and non-infected cecal tissue: CCR5P-PLC-PKC signaling pathway.

				4 hours pi		8 hours pi		24 hours pi	
Functions	Peptide name	UniProt ID	p-site*	Fold change	*p*-value	Fold change	*p*-value	Fold change	*p*-value
**Degranulation** **NO induction** **Migration** **ROS production**	CCR5	P51681	S276	1.412	0	1.088	0.03	1.234	0
	PKCA	P1752	S659 T640	1.35 2.09	0.001 0	- -	- -	- -	- -
	INOS	P35228	Y148	1.263	0	-1.537	0	–	–
	FAK	Q05397	Y397	1.220	0.001	-1.263	0	-1.143	0.001
	CRK	P46108	Y252	1.806	0.001	–	–	-1.402	0
	PYK2	Q14289	Y405	1.259	0.001	–	–	-1.402	0
	NCF10 (p47phox)	P14598	S360	1.086	0.029	1.170	0.03	–	–

Selection of peptides differentially phosphorylated between non-infected group and S. Enteritidis challenge group in the cecum. The UniProt ID identifies the protein, while the p-site identifies the specific phosphorylation target site on that protein. Fold-Change indicates the directionality of phosphorylation status for each treatment compared to control. The p-value is the measure of significance (α = 0.05). UniProt IDs and p-site correspond to human proteins for annotation purposes.

*= Phosphorylation site.

#### Apoptosis Signaling Pathway

Early infection of the chicken cecum by *S*. Enteritidis appears to trigger apoptosis *via* both intrinsic and extrinsic pathways (**Table 7**; **Supplemental Figure 4**). Both the Fas and TNFR1 death receptors were activated by phosphorylation by 8 h post-infection. The extrinsic pathway *via* Fas is stimulated by the binding of the Fas ligand to the Fas receptor which the phosphorylated FADD (Fas-associated death domain) which activates (phosphorylates) the initiator caspase, caspase 8 (CASP8). CASP8 the activates (phosphorylates) caspase 3 that results in the subsequent apoptosis. Remarkably, the TNFR1 receptor appears to activate both the extrinsic and intrinsic apoptosis pathways during early infection of the cecum by *S*. Enteritidis. Like the Fas receptor, TNFR1 activates the extrinsic pathway by activating the FADD →CASP8→CASP3 cascade. TNFR1 also activates the intrinsic pathway by activating (phosphorylating) the RIPK1 (receptor interacting serine/threonine protein kinase 1) and TRAF2 (TNF-R associated 2) adaptor protein complex. This complex phosphorylates ASK (Apoptosis signal-regulating kinase 1) which activates c-Jun N-terminal kinase (JNK). JNK then activates the two pro-apoptotic proteins BAD (BCL2 associated agonist of cell death) and BID (BH3 interacting-domain death agonist) which initiate apoptosis by forming a pore in the mitochondrial outer membrane that allows cytochrome c to escape into the cytoplasm and activate the pro-apoptotic caspase cascade. Lastly, the X-linked inhibitor of apoptosis protein (XIAP), whose function when phosphorylated, is to inhibit apoptosis was, in fact, dephosphorylated in the *S*. Enteritidis-infected tissue. The dephosphorylation of XIAP blocks the activation of an apoptosis inhibitory pathway during *Salmonella* infection.

**Table 7:** Members of the apoptosis signaling pathway showing differential phosphorylation between *Salmonella enterica* serovar Enteritidis-infected cecal tissue and non-infected cecal tissue.

			4 hours pi		8 hours pi		24 hours pi	
Peptide name	UniProt ID	p-site*	Fold change	*p*-value	Fold change	*p*-value	Fold change	*p*-value
**TRAF2**	Q12933	T117	1.091	0.027	1.178	0.0003	1.091	0.031
**CHUK**	Q015111	S194	1.086	0.017	–	–	1.262	0.002
**XIAP**	P98170	S87	-1.752	0.00001	–	–	-1.312	0.010
**cFLAR**	O15519	Y283	1.392	0.00004	–	–	1.191	0.0009
**CASP6**	P55212	S268	2.14	0.000	1.325	0.0011	–	–
**CASP8**	Q14790	Y383	1.335	0.0003	1.095	0.035	1.305	0.001
**CASP8**	Q14790	S350	–	–	1.095	0.014	1.225	0.003
**BAD**	Q92934	S112	1.180	0.00002	1.05	0.044	1.110	0.018
**NTRK1** **(TrkA)**	P04629	Y785	-1.368	0.004	-1.471	0.0006	-1.192	0.039
**RIPK1**	Q13546	Y384	1.170	0.047	1.151	0.001	–	–
**FAS**	P25445	Y265	–	–	1.316	0.0004	1.295	0.002
**RelA** **(NF-κB p65)**	Q04206	S281	1.098	0.041	1.294	0.0002	1.116	0.0007
**CASP3**	P42574	S158	–	–	1.173	0.0002	1.161	0.045
**BID**	P55957	Y56	1.008	0.046	1.029	0.033	1.122	0.037
**TNF-R1**	P19438	Y343	–	–	1.355	0.002	1.196	0.041
**ASK-1**	Q59GL6	S852	–	–	1.203	0.008	1.158	0.011
**JNK**	P45938	Y185	1.402	0.000	–	–	1.201	0.023

Selection of peptides differentially phosphorylated between non-infected group and S. Enteritidis challenge group in the cecum. The UniProt ID identifies the protein, while the p-site identifies the specific phosphorylation target site on that protein. Fold-Change indicates the directionality of phosphorylation status for each treatment compared to control. The p-value is the measure of significance (α = 0.05). UniProt IDs and p-site correspond to human proteins for annotation purposes.

*= Phosphorylation site.

#### JAK-STAT Signaling Pathway

The Janus kinase (JAK)/signal transducer and activator of transcription (STAT) signaling pathway is a mutual conduit of many cytokine signal transductions involved in cell proliferation, apoptosis, differentiation, and inflammatory response. The JAK-STAT pathway is fundamental for inhibiting the inflammatory response, initiating innate immunity, and coordinating adaptive immune mechanisms (O’Shea et al., 2015). Within the first 24 hours post-infection, a differentiated series of phosphorylation events occurred at the cytokine receptor level in the *S*. Enteritidis-infected birds when compared to the non-infected control birds (**Table 8**). First, a significant increase in phosphorylation of the interferon-alpha receptor (IFNAR1), interferon-gamma receptor (IFNGR1), interleukin-2 receptor (IL2RB), and IL6ST (gp130) (**Table 8**). Simultaneously, there is a significant decrease on the phosphorylation of the IL-4 receptor (IL-4R) at all time points. Interestingly, there was no change in the phosphorylation of the IL-7 receptor (IL-7R) from the non-infected controls at 4 and 8 h pi, but was significantly dephosphorylated at 24 h p.i. The IL10R was only significantly dephosphorylated at 4 h pi with no change from the control at 8 and 24 h p.i. (**Table 8**).

**Table 8:** Members of the JAK-STAT signaling pathway showing differential phosphorylation between *Salmonella enterica* serovar Enteritidis-infected cecal tissue and non-infected cecal tissue.

			4 hours pi		8 hours pi		24 hours pi	
Peptide name	UniProt ID	p-site*	Fold change	*p*-value	Fold change	*p*-value	Fold change	*p*-value
**IFNGR1**	P15260	Y416	1.652	0	1.450	0	–	–
**IFNAR1**	P17181	S547 Y486	1.408 -	0 -	1.305 -1.468	0 0	1.155 1.166	0.01 0.05
**JAK1**	P23458	Y1028	–	–	–	–	-1.19	0.002
**JAK2**	O60674	Y1004 Y219	1.114 1.118	0.01 0.01	- 1.512	- 0	-1.165 -	0.03 -
**JAK3**	P52333	Y980 Y938	1.135 1.302	0.03 0.02	1.185 -	0.02 -	1.217 1.145	0 0.01
**TYK2**	P29697	Y35	-2.502	0	–	–	-1.451	0.03
**STAT1**	P42224	S729	-1.61	0.01	–	–	-1.19	0.001
**STAT3**	P40763	Y706 S728	-2.40	0.002	-2.235	0.002	–	–
**STAT4**	Q14765	S722 Y694	-1.234 -2.236	0.05 0.002	- -	- -	1.041 -	0.04 -
**STAT5B**	P51692	Y740 Y699	2.207 -1.60	0.001 0	1.073 -	0.03 -	1.514 -1.09	0.01 0.002
**STAT6**	P42226	Y236	-1.205	0.001	-1.173	0.05	–	–
**IL2RB**	P14784	Y464 Y270	1.75 -	0 -	1.09 -1.102	0.02 0.004	1.57 -	0 -
**IL10RA**	Q13651	Y475	-1.178	0.01	–	–	–	–
**IL4R**	P24394	Y230	-1.641	0	-1.285	0.03	-1.24	0.01
**IL7R**	P16871	Y454	–	–	–	–	-1.169	0.001
**IL6ST** **(gp130)**	P40189	Y905 Y764	1.271 1.30	0.02 0.0001	- 1.321	- 0	- 1.104	- 0.002
**SOCS3**	O14543	Y205	-1.428	0	-1.282	0.0002	-1.343	0.0003
**EP300** **(HAT)**	Q99558	S104 S89	1.17 -	0.0003 -	-1.326 -	0 -	- -	- -
**STAM**	Q92783	Y527	-1.37	0.0003	1.166	0.02	1.22	0
**PIM1**	P11309	Y218	1.256	0	-1.351	0	–	–
**CCND1** **(cyclin1)**	P29385	T283	1.112	0.04	–	–	1.114	0.01
**GRB2**	P62993	Y209	–	–	-1.129	0.001	–	–
**AKT1**	P31749	T308 S473	-1.121 1.245	0.03 0	- 1.126	- 0.03	- 1.361	- 0
**AKT3**	Q94243	S472 T305	- -1.094	- 0	1.175 -	0.02 -	- -1.154	- 0.004
**PIK3R1**	P27986	S608 Y528 Y556	1.908 -1.419 -	0 0.0002 -	1.107 1.163 1.71	0.01 0.002 0.01	- - -	- - -
**PIK3R2**	O00459	Y612 Y471	1.67 -	-.01 -	1.521 1.150	0.04 0.05	- -	- -
**CBL**	P22681	Y684 Y728 Y773	1.611 1.090 1.391	0 0.01 0	- - 1.115	- - 0.03	1.199 1.353 -	0.04 0 -
**SOS1**	Q07889	S1208 S1234	1.71 -	0.005 -	1.42 -1.04	0.006 0.05	-1.199 -	0.04 -

Selection of peptides differentially phosphorylated between non-infected group and S. Enteritidis challenge group in the cecum. The UniProt ID identifies the protein, while the p-site identifies the specific phosphorylation target site on that protein. Fold-Change indicates the directionality of phosphorylation status for each treatment compared to control. The p-value is the measure of significance (α = 0.05). UniProt IDs and p-site correspond to human proteins for annotation purposes.

*= Phosphorylation site.

Early infection of the cecum by *S*. Enteritidis appears have differential effects in the JAK kinases. JAK1 and TYK2 are targets for dephosphorylation at 4 h and 4-24 h p.i., respectively; whereas JAK2 and JAK3 were significantly phosphorylated by *S. Enteritidis* infection when compared to the non-infected controls (**Table 8**).

Additionally, early infection of the cecum by *S*. Enteritidis appears to target most STAT proteins (STAT1, STAT3, STAT4, STAT6) which were all significantly dephosphorylated during the first 24 h p.i. The lone exception was STAT5B which was significantly phosphorylated at Tyr749 at all three early post-infection time points following infection with *S*. Enteritidis.

It should also be noted that the suppressor of cytokine signaling 3 (SOCS3) was dephosphorylated during the entire initial 24 hours of *S*. Enteritidis infection.

#### T Cell Receptor Signaling Pathway

T cells need two signals to become fully activated. Signal 1 is provided by the T-cell receptor when recognizing a specific antigen associated with major histocompatibility complex molecules. Signal 2 comes from co-stimulatory receptors such as CD28, presented on the surface of other immune cells. It is expressed only when an infection was detected by the innate immune system. Engagement of the T cell receptor through these 2 signals results in a series of signaling cascades that lead to T-cell proliferation, cytokine production and differentiation into effector cells (Smith and Gobel, 2022). These protein cascades lead to the activation of transcription factors. Transcription factors involved in T cell signaling pathway are the NFAT, NF-κB and AP1 (Smith and Gobel, 2022). Specifically, TCR activation results in the phosphorylation and activation of the lymphocyte-specific protein tyrosine kinase. Phosphorylation of Lck can lead to the activation of two transcription factors, NFAT *via* phospholipase C and AP-1 through MAPK signaling pathway (Salmond et al., 2009; Smith-Garvin et al., 2009; Nika et al., 2010; Van der Merwe and Dushek, 2011). The third transcription factor, NF-kB can be activated through the stimulation of the co-stimulatory molecule CD28 *via* the phosphoinositide 3-kinase (PI-3K) pathway (Smith and Gobel, 2022).

Analysis of the T cell receptor signaling pathway revealed several significant changes in phosphorylation events (**Table 9**):

**Table 9:** Members of the T cell receptor signaling pathway showing differential phosphorylation between *Salmonella enterica* serovar Enteritidis-infected cecal tissue and non-infected cecal tissue.

			4 hours pi		8 hours pi		24 hours pi	
Peptide name	UniProt ID	p-site*	Fold change	*p*-value	Fold change	*p*-value	Fold change	*p*-value
**HRAS**	P01112	T35	1.695	0.001	1.260	0.001	–	–
**CBL**	P22681	Y684 Y728 Y773	1.611 1.090 1.391	0 0.01 0	- - 1.115	- - 0.03	1.199 1.353 -	0.04 0 -
**MAPK3** **(ERK1)**	P27361	T193	1.160	0.03	1.194	0	–	–
**MAP3K8** **(COT)**	P41279	S400 T290	-1.476 -1.166	0 0.004	1.248 1.104	0.002 0.002	-1.301 -	0 -
**PRKCQ** **(PKC θ)**	Q04759	T546	-1.386	0	1.189	0.002	-1.136	0.01
**MAPK14** **(p38 α)**	Q16539	Y323 Y181	1.314 -	0.005 -	1.136 -	0.04 -	1.228 -	0.03 -
**CHUK** **(IκκA)**	O15111	S194 T34	1.086 -	0.02 -	- 1.191	- 0.005	1.262 -	0.002 -
**MAP3K7** **(TAK1)**	O43318	T177 S446	- 1.160	- 0.006	1.365 -	0.02 -	- 1.215	- 0.001
**MAP2K1** **(MEK1, MKK1)**	Q02750	S222 S300	-1.280 1.133	0.001 004	1.140 1.230	0.03 0.0003	- 1.105	- 0.02
**ITK**	Q08881	Y191 Y523	- 1.357	- 0.002	- 1.164	- 0.01	-1.408 1.194	0 0.02
**LCK**	P06289	Y504 Y393	- -1.306	- 0	-1.070 1.090	0.04 0.05	-1.230 -1.120	0.001 0.02
**AKT1**	P31749	T308 S473	-1.121 1.245	0.03 0	- 1.126	- 0.03	- 1.361	- 0
**AKT3**	Q94243	S472 T305	- -1.094	- 0	1.175 -	0.02 -	- -1.154	- 0.004
**NFκB1A** **(IκB)**	P25963	Y46 S36 S194	1.193 - 1.086	0.04 - 0.02	1.178 - -	0.01 - -	- - 1.262	- - 0.003
**RAF1**	P04049	S43 S343 S259	- 1.219 -	- 0.003 -	- - 1.297	- - 0.03	- -1.10 -	- 0.001 -
**PIK3R1**	P27986	S608 Y528 Y556	1.908 -1.419 -	0 0.0002 -	1.107 1.163 1.71	0.01 0.002 0.01	- - -	- - -
**PIK3R2**	O00459	Y612 Y471	1.67 -	0.01 -	1.521 1.150	0.04 0.05	- -	- -
**FOS**	P01100	S349	1.199	0.004	–	–	1.187	0.005
**NFκB1** **(NFκB p105)**	P19838	S943 S938 S342	-1.583 - 1.803	0 - 0	- - 1.233	- - 0.004	- - -	- - -
**NFκB1** **(NFκB p100)**	Q00653	S818 S872	1.453 1.926	0 0	-1.433 1.214	0 0.02	- -	- -
**Calmodulin**	P62158	Y100 T80	- -	- -	- -1.122	- 0.002	- -	- -
**NFATC1**	Q13469	S225 S175	1.253 1.706	0.04 0.007	-1.118 1.264	0.02 0.03	- -	- -
**NFATC2**	O95647	S526 S351 S302	- 1.79 1.821	- 0.001 0.001	-1.223 1.241 1.16	0 0.03 0.04	1.298 - -	0 - -
**NFATC3**	Q12968	S203	1.32	0.0004	-1.439	0	–	–
**PLCG1**	P19174	Y717 Y675	- -1.251	- 0	-1.245 -	0.003 -	-1.122 -1.205	0.02 0.006
**SOS1**	Q07889	S1208 S1234	1.71 -	0.005 -	1.42 -1.04	0.006 0.05	-1.199 -	0.04 -
**CD28**	P10747	Y192	1.368	0	–	–	1.111	0.03
**JUN**	P05412	Y59 Y69	-1.452 1.138	0.001 0.03	- 1.178	- 0.03	-1.688 -	0 -
**CDC42**	P60953	Y64	1.42	0	1.218	0.005	–	–
**GSK3β**	P49884	Y351 S144	- -1.38	- 0	-1.188 1.347	0.003 0	- -	- -
**PAK1**	Q13153	T422 S198 T212	- -1.28 1.442	- 0.02 0.02	- 1.332 1.275	- 0 0.04	1.195 1.071 -	0.001 0.05 -

Selection of peptides differentially phosphorylated between non-infected group and S. Enteritidis challenge group in the cecum. The UniProt ID identifies the protein, while the p-site identifies the specific phosphorylation target site on that protein. Fold-Change indicates the directionality of phosphorylation status for each treatment compared to control. The p-value is the measure of significance (α = 0.05). UniProt IDs and p-site correspond to human proteins for annotation purposes.

*= Phosphorylation site.

First, the three transcription factors (NFAT, AP-1, and NF-κB) exhibited differential phosphorylation activity within 4-8 h post infection with *S*. Enteritidis with a return to control levels by 24 h (**Table 9**). The significant phosphorylation of the members of the NFAT family n the ceca of *S*. Enteritidis-infected chickens (**Table 9**) is a meaningful finding because inactivated NFAT proteins in the cytoplasm of a cell are in their phosphorylated form. Following T cell receptor (TCR) stimulation, cytoplasmic NFAT proteins are dephosphorylated and translocate from the cytoplasm to the nucleus where they regulate transcription of key cytokine genes. Thus, based on the findings here the increased phosphorylation of NFAT prevents the protein from translocating to the nucleus. The significant phosphorylation of the adapter protein GRB2, SOS, and the MAPKs (MEK1, ERK1, and p38) and the JUN and FOS proteins demonstrates the activation of the transcription factor, AP-1. AP-1 controls a number of cellular processes including differentiation, proliferation, and apoptosis (Karin et al., 1997). Lastly, The Ser/Thr kinase COT, CHUK (Iκκ-A), NF-κB1A (IκB), and NF-κB (p105 and p100), all significantly phosphorylated within 4-8 h of infection with S. Enteritidis (**Table 9**), but all were found to be no different than non-infected control levels by 24 h (**Table 4**). Both NFAT and NF-κB are transcription factor that when phosphorylated can regulate genes responsible for both the innate and adaptive immune responses when activated by various intra- and extra-cellular stimuli then translocate into the nucleus and stimulates the expression of genes involved in a variety of immune functions (Zanoni and Granucci, 2012; Banoth et al., 2015). Secondly, phospholipase C-1 (PLCG1) and calmodulin were significantly dephosphorylated in the *S*. Enteritidis-infected cecal tissue by 4 hours post-infection when compared to the non-infected control cecal tissue. The importance of this is twofold: PLCG1 generates second messenger molecule inositol 1,4,5-trisphosphate (IP3) (Essen et al., 1997). IP3 binds to calcium channel receptors on the endoplasmic reticulum (ER) induces the release of calcium (Ca^2+^) into the cytosol of the T cell.

## Discussion

Understanding both sides of the host –*Salmonella* interaction is essential in discovering alternative strategies to manage infections in poultry. The host’s antimicrobial immunity and the bacteria’s evasion of that immunity both rely on appropriate regulation of transcriptional and signaling networks. To date, the majority of the studies in the chicken-*Salmonella* interaction has been microbe-centric, concentrating on the pathogen’s infection of the bird intestine by using the *Salmonella* pathogenicity islands 1 and 2 (SPI-1 and SPI-2) that encode a specialized type III secretion system (T3SS) to secrete multiple protein effectors into the intestinal epithelium which manipulate the host cell biology to aid in bacteria invasion, intracellular survival, and to modulate host immune responses (Barrow et al., 2012; Figueira and Holden, 2012; Wigley, 2014; Eng et al., 2015). On the host side of the interaction, the transcriptional and signaling descriptions of the interaction has concentrated on the post-infection period starting 2 days *after* the initial infection by the pathogen. This breach results in a directed innate immune response to purge the bacterium and includes the release of antimicrobial peptides (Crhanova et al., 2011; Garcia et al., 2020), activation of pattern-recognition receptors (Keestra et al., 2013, Kogut, 2022a; Kogut, 2022b), release of chemokines and cytokines (Withanage et al., 2005; Bernd et al., 2007; Rychlik et al., 2014; Meijerink et al., 2021), and recruitment of a variety of immune cells (Van Immerseel et al., 2003; Bernd et al., 2007; Pieper et al., 2011; Meijerink et al., 2021).

Therefore, what is needed is more insight into the immediate response of the chicken to the *Salmonella* infection process (first 24 hours). For example, chickens infected with *Salmonella* do not develop clinical disease and the acute inflammatory response is short-lived (Wigley, 2014). Thus, it is vital to understand how the chicken responds to a *Salmonella* breach of the mucosal barrier system and what is involved in the regulation of the intestinal response that prevents excessive intestinal inflammation and damage yet does not eliminate pathogen colonization. Our recent development of chicken-specific peptide arrays for kinome analysis of host phosphorylation-based cellular signaling responses provided us with the opportunity to develop a more detailed understanding of the chicken host-pathogen interactions with *Salmonella* (Arsenault and Kogut, 2015; Perry et al., 2020). We have used this technology to describe the immunometabolic phenotypic changes in the avian cecum of *Salmonella*-infected chickens that decreased the host responsiveness resulting in the establishment of persistent colonization starting around 4 days after the initial infection (Kogut et al., 2016).

Overall, in all the pathways described here, the majority of the differential phosphorylation events between the infected cecal tissue compared to the non-infected control tissue occurred with the first 4-8 hours post-infection. In general, from 8-24 hours post-infection, there was a decrease in phosphorylation alterations in the infected tissue with a return to control levels of phosphorylation. Based on the signaling pathways that were differentially activated (TLR, NOD, chemokine, apoptosis, JAK-STAT), the chicken responds very rapidly with an orchestrated innate response: recognition (TLR and NOD signaling pathway), attraction of activated immune cells (chemokine and JAK-STAT signaling pathways), and the prevention/reduction in pathogen numbers (apoptosis).

Based on the findings described here, the chicken pattern recognition receptors, Toll-like receptors (TLR) and nucleotide-binding oligomerization domains receptors (NOD), recognized molecular associated microbial patterns (MAMPs) associated with *S*. Enteritidis (**Tables 4**, **5**; **Supplemental Figures 1** and **2**). The activity of both pathways was activated (phosphorylated) 4-8 hours post-infection and their activity decreased by 24 post-infection. Pathways analysis suggests that NOD1 and NOD2 are specifically involved in the immediate recognition of a *Salmonella* in chickens. NOD1 and NOD2, two prototypic NLRs, sense the cytosolic presence of bacterial peptidoglycan fragments that escaped from endosomal compartments, driving the activation of NF-κB and MAPK, cytokine production and apoptosis (Li et al., 2017; Tao et al., 2017). However, NOD2 appears to be absent in birds (Boyle et al., 2013). There is no evidence, based on the pathway analysis, that inflammasome-mediated pathways were involved the chicken recognition of *S*. Enteritidis.

Although kinome analysis of the *S*. Enteritidis-infected tissue does not provide the specific TLR that initiated the TLR signaling pathway in the chicken, previous studies have convincingly shown that recognition of flagellin by TLR5 is the primary recognition receptor that stimulates the bird’s innate response (Iqbal et al., 2003). Is also possible that TLR4 that recognizes LPS on the *Salmonella* cell wall and TLR21 which recognizes CpG (bacterial unmethylated DNA) could be involved in initiating the TLR signaling pathway as both have been found in chickens (Higgs et al., 2006). However, chickens are very refractory to LPS except at high levels not found in bacterial cell walls (Adler and DaMassa, 1979; Keestra and Van Putten, 2008).

Chemokines are small chemoattractant peptides that provide directional cues for the cell trafficking and thus are vital for protective host response. In addition, chemokines regulate several biological processes of hematopoietic cells that lead to cellular activation, differentiation and survival. The chemokine signal is transduced by chemokine receptors expressed on the hematopoietic immune cells. After receptor activation, several diverse downstream pathways are activated which encompass a number of biological activities of hematopoietic cells that lead to cellular activation, differentiation and survival. Based on the findings here, the kinomic analysis of the chemokine signaling pathway demonstrates the activation of three downstream pathways: (1) the MAPK/JAK-STAT cascades involved in cell migration, cytokine production, and cell survival, (2) the PI3K → AKT→ Itk cascade that regulates leukocyte transmembrane migration and actin reorganization, and (3) the CCL5 → PLC → PKC cascade that causes the induction of nitric oxide, production of reactive oxygen species, and degranulation (Hughes et al., 2001; Wang et al., 2005; Sick et al., 2006; Hughes et al., 2007). All three cascades were highly phosphorylated in the 4 h post-infection cecal tissue when compared to the non-infected control tissue. From 8-24 h post-infection, there is a dramatic increase in either dephosphorylation events or no differences from the control inn the *S*. Enteritidis-infected tissues. The kinome analysis of the timing of the chemokine signaling pathway coincides with our previous studies where we found that paratyphoid *Salmonella* serovars induced a rapid (within 4 h) inflammatory response characterized by an influx of heterophils (the primary granulocytic cell the gastrointestinal tract of chickens (Kogut et al., 1993; Kogut et al., 1994; Genovese et al., 1999; Swaggerty et al., 2006).

Apoptosis is a genetically regulated process of caspase-dependent form of cell death of damaged or infected cells in response to intrinsic or extrinsic signaling cascades (Galluzzi et al., 2018; Wemyss and Pearson, 2019). Typically, in the intestine apoptosis of epithelial cells from the mucosa occurs with little, if any, inflammation and disruption of the epithelial barrier integrity (Schwerk and Schultze-Osthoff, 2005). However, *Salmonella*-induced cell death (apoptosis, necroptosis, pyroptosis) are crucial components of bacterial-mediated gastroenteritis in mammals (Kim et al., 1998; Fink and Cookson, 2007; Hefele et al., 2018; Wemyss and Pearson, 2019). Alternatively, the major paratyphoid *Salmonella* serovar infections of poultry leads to little or no clinical signs of disease (Barrow et al., 2012) which may coincide with the fact that there have been no reports of *Salmonella*-induced cell death in the chicken intestine during colonization and infection. Based on the findings here, *S*. Enteritidis infection of the cecum induces the apoptosis signaling pathway when compared to the non-infected control cecal tissue. More specifically, in fact, based on the alterations in phosphorylation, *S* Enteritidis infection stimulated both intrinsic and extrinsic apoptotic signaling cascades (**Table 7**; **Supplemental Figure 4**). The lack of clinical signs of disease in chickens during paratyphoid *Salmonella* infection, the importance of maintaining both intestinal homeostasis and the intestinal barrier during the initial phase of infection, strongly suggests that the apoptotic pathway outlined here is a host defense strategy to limit bacterial replication and survival (Sellin et al., 2014). Limiting the initial infection and replication of the pathogen may be an important component of the ‘disease tolerance’ mechanism of host defense that is unique to the chicken-*Salmonella* interaction that we have described previously (Kogut and Arsenault, 2017; Lee et al., 2020). Finally, we found no evidence, based on phosphorylation events, of either of the pro-inflammatory forms of cell death, necroptosis nor pyroptosis in the *Salmonella*-infected cecal tissues. Specifically, necroptosis is an inflammatory-mediated, caspase-independent cell death mechanism. We have shown clearly (**Table 7**; **Supplemental Figure 4**) that caspases 3, 6, and 8 are all phosphorylated; thus activated as part of the extrinsic apoptosis pathway. Further, pyroptosis is a highly inflammatory cell death pathway that requires the development of an inflammasome which mediates the event. We found no evident of an inflammasome development in our studies.

Further kinome pathway analysis of the S. Enteritidis-infected cecal tissue revealed the differential phosphorylation of individual peptides in the JAK-STAT pathway (**Table 8**). The JAK-STAT pathway is a signaling cascade that provides a direct mechanism to translate an extracellular signal into a transcriptional response, in S. Enteritidis-infected cecal tissue. The JAK-STAT system consists of three main components: (1) a receptor; (2) Janus kinase (JAK); and (3) Signal Transducers and Activator of Transcription (STAT) (Hu et al., 2021). Based on the results from the present experiment (**Table 8**), the IFN-α, IFN-γ, IL-2, and IL-6 cytokine family (gp130) receptors were phosphorylated; whereas, IL-4, IL-10 were dephosphorylated, and IL-7 receptor showed no change from the non-infected control cecal tissue, at 4 and 8 hours after infection, but was dephosphorylated at 24 h post-infection. IFN-γ is characteristic of a Th1 response whereas IL-2 is normally produced by T cells during an immune response and involved in growth, proliferation, and differentiation of T cells to become “effector” T cells (Cantrell and Smith, 1984; Smith, 1988). Traditionally, IL-6 is a pro-inflammatory cytokine involved in stimulating an immune response during infection which may very well be the case with these findings. However, we also consider strong evidence that IL-6 is involved in metabolic function, rather than just immune response. IL-6 induction has been found to stimulate a metabolic reprogramming [Pavlov and Tracey (2012); Flint et al., 2016 and Ghanemi and St Amand (2018)] which could be happening here with the initiation of an innate response to *Salmonella* infection. *Salmonella* infection activates the innate immune system as described above but activating the innate immune system places considerable energy and resource demands on the chicken that requires amplified metabolic requirements and nutrient consumption (Klasing, 2007; Klasing, 2017). The increased phosphorylation of the IL6ST (gp130) receptor throughout the initial 24 hours of infection with *Salmonella* may be an immunometabolic signature of the instigation of the innate immune response.

Neither the IL-4R nor the IL7R were activated (phosphorylated) during the first 24 h after *S*. Enteritidis infection in the cecum when compared to the non-infected control tissues. Since IL-4 normally induces differentiation of naïve T helper cells to Th2 cells and IL-7 is involved in early T cell development, it is undoubtedly too early in the immune process for responses, the results are suggestive of premature involvement of T cell-mediated immune responsiveness so early in the infection process. Interestingly, the IL2RB was phosphorylated throughout the 24 hr initial infection period. Classically, IL-2 was described as a T cell growth factor and to also stimulate growth promoting activity in B cells and NK cells (Henney et al., 1981; Robb et al., 1981; Waldmann et al., 1984). However, we showed 20 years ago that IL-2 can directly activate chicken heterophils to exert effector functions and induce heterophil activation (Kogut et al., 2002). It has subsequently been proven that IL-2 has functional activity that connects innate and acquired immunity (reviewed Bendickova and Fric, 2020).

Both the type I interferon (IFNAR1, IFN-α) and type II interferon receptors (IFNGR1, IFN-γ) were phosphorylated 4-8 hours after cecal infection with *S*. Enteritidis. Traditionally, IFN-α is an antiviral cytokine, but recent evidence suggests that it is involved further in the development of innate immunity by playing a role in restricting bacterial infections, including Salmonella (Alphonse et al., 2021). In fact, further evidence is that IFN-α signaling can be regulated by the NOD-1 receptor (Kienes et al., 2021). We can speculate that the sensing of the *S.* Enteritidis infection by NOD-1, as described above, can lead to the production of IFN-α that controls the level of colonization and infection by the pathogenic bacteria in the chicken cecum. We have further shown that IFN-γ can activate *in vitro* heterophil functional activity against *Salmonella* (Kogut et al., 2001; Kogut et al., 2005) and the presence of IFN-γ and IL-2 in a *S*. Enteritidis immune cytokine cocktail protects day-old chicks against *Salmonella* infections mediated by activated heterophils (Kogut et al., 1996; Verduzzo et al., 2009).

The dephosphorylation of SOCS3 suggests that *Salmonella* virulence factors are not directly involved in the inhibition of cytokine signaling during the early infection period in the cecum. Alternatively, the host is initiating the innate response necessary to fight infection through the release of cytokines and that the activation of the negative regulator of cytokine signaling is not required at this time.

Cytokine receptor proteins lack enzymatic activity, thus are dependent upon JAKs to initiate signaling upon binding of their ligands. The JAK family has four members: JAK1, JAK2, JAK3 and tyrosine kinase 2 (TYK2) (Hu et al., 2021). The STAT family consist of seven members: STAT1, STAT2, STAT3, STAT4, STAT5a, STAT5b, and STAT6 (Hu et al., 2021; Nobel et al., 2021). We observed a selective activation of the JAK proteins with JAK2 and JAK3 the only JAK family members that were phosphorylated in the ceca from the *S*. Enteritidis-infected chickens (**Table 8**). JAK2 signaling is activated by interferon receptors and the gp130 receptor family (IL-6R) and is an essential tyrosine kinase for initiating the innate immune response (Pena et al., 2010). JAK3 is predominantly expressed in hematopoietic lineage and intestinal epithelial cells, but its role in cytokine signaling is more restricted than other JAKs with IL-2R, IL-4R, and IL-7R being the most common receptors (Kumar et al., 2007; Mishra et al., 2012). In the intestinal epithelium, IL-2 induces activation of Jak3 to facilitate/maintain mucosal homeostasis including mucin secretion and tight junction proteins (Kumar et al., 2021).

STAT5b is the sole member of the STAT family that is phosphorylated in the cecum during the initial infection process (4-24 hours) by *S*. Enteritidis. JAK-STAT5B signaling in the intestine is involved in the host response to inflammation and infection (Nobel et al., 2021). In the intestine, STAT5B activation is regulated by specific members of IL-2 family of cytokines (Awasthi et al., 2021) and IL-6 (Jiang et al., 2009; Rani and Murphy, 2016) is involved in multiple intestinal physiological responses including mucosal barrier function through tight junction permeability, mucin production, and regeneration and proliferation of intestinal epithelial stem cells (Han et al., 2009; Gilbert et al., 2015; Karin and Clevers, 2016; Degirmenci et al., 2018). However, activated STAT5 is short-lived and undergo rapid deactivation (Grimley et al., 1999). Combined with the cytokine receptor phosphorylation data discusses previously (IL2R and IL6R), it is reasonable to assume that STAT5B phosphorylation found here is involved in maintaining the cecal function during the initial *Salmonella* infection stage.

Stimulation of the T cell receptor results in the activation of the TCR signal transduction pathway. This pathway can activate the transcription factors nuclear factor κB (NF-κB), nuclear factor of activated T-cells (NFAT), and activator protein 1 (AP-1), that induce expression of cytokine genes (Smith-Garvin et al., 2009). However, based on up-stream regulators, neither NFAT nor NF-κB are activated through the T cell receptor pathway. First, the NFAT transcription factor family are regulated by calcium signaling which is critical to activation of NFAT because calmodulin activates the serine/threonine phosphatase calcineurin. Nuclear import of NFAT is dependent on the calcium level inside of a cell. If the calcium level drops, the exporting kinases in a nucleus rephosphorylate NFAT causing the transcription factor to revert into its inactive state and is exported back to the cytosol where maintenance kinases finish the rephosphorylation to keep it in the inactivated state (Baba and Kuroski, 2016; Park et al., 2020). Here we found a significant dephosphorylation in calmodulin, meaning that there is a dramatic reduction in calcium levels in the *S*. Enteritidis-infected tissue thus inhibiting NFAT activity. Second, NFAT requires the activity of PLC-1, which generates the second messengers, diacylglycerol (DAG) and inositol 1,4,5-trisphosphate (IP3). IP3 induces an increase in the concentration of cytoplasmic calcium (Ca^2+^) and activation of the Ca^2+^-dependent phosphatase calcineurin, which results in the rapid activation of NFAT, which is followed by its translocation to the nucleus (Baine et al., 2009). Thus, the dephosphorylation of PLCG1 prevents NFAT activation due to the decrease in intracellular calcium transport into the immune cells. Likewise, the dephosphorylation of PLCG1 inhibits the generation of DAG which is a vital second messenger in NF-κB activation *via* the T cell receptor. However, activation of NF-κB by PRRs in the innate response does not require PLCG1 generation of the DAG molecule to activate downstream proteins. PRRs use MyD88-dependent pathways to activate NF-κB activity (Akira et al., 2006, Kumar et al., 2011).

One intriguing finding from the current studies that links with our previous report on Salmonella persistence is the consistent dephosphorylation of phospholipase cγ1 (PLCG1) enzyme in the S. Enteritidis -infect cecal tissue (Kogut et al., 2017). This ‘targeting’ of PLCG1 by Salmonella seems to be a feature of infection between 24-96 hours post-infection. Even though we are not aware of what bacterial factors may be involved in dephosphorylating PLCG1, it is reasonable to assume that this action is part of the asymptomatic inflammatory response of chickens that leads to the development of a persistent Salmonella infection in the chicken. Further it appears possible that Salmonella inhibiting PLCG1 phosphorylation is part of the unique survival strategy of paratyphoid *Salmonella* in poultry that minimizes host defenses mechanisms (disease resistance) during the initial infection and then further uses the inhibition of PLCG1 activity as part of the immunometabolic reprogramming that converts host defense strategy to disease tolerance. Disease tolerance is the ability of the host to limit the damage caused by both the pathogen and the host immune response, i.e., immunopathology (Ayres and Schneider, 2012). PLCG1 would be a logical focal point of the initial *Salmonella* colonization/infection repertoire since it is involved in regulating innate immune functions including phagocytosis, the oxidative burst, cell migration and TLR-mediated signaling (Bae et al., 2017; Zhu et al., 2018; Jing et al., 2021).

## Data Availability Statement

The raw data supporting the conclusions of this article will be made available by the authors, without undue reservation.

## Ethics Statement

These studies were approved by the Animal Care and Use Committee (ACUC) at the Southern Plains Agricultural Research Center, Agricultural Research Service, United States Department of Agriculture (ACUC #2012007), which meets all federal requirements as defined in the Animal Welfare Act, the Public Health Service Policy, and the Humane Care and Use of Laboratory Animals.

## Author Contributions

All authors contributed to the conception and design of the study. MK, KG, JB, HH, and CS conducted the infection studies and samples collections. RA conducted the kinome array and MK and RA conducted the bioinformatic analysis of the kinome data. YF and RA conducted the statistical analyses of all the data. MK wrote the first draft of the manuscript. All of the authors contributed to the manuscript revision and read and approved the submitted version.

## Funding

The research was conducted as part of the ARS CRIS Project: ‘Immunological and Practical Approaches to Manipulate the Ecological Niches and Reduce Foodborne Pathogens in Poultry’ Project #3091-32000-037-00D).

## Conflict of Interest

The authors declare that the research was conducted in the absence of any commercial or financial relationships that could be construed as a potential conflict of interest.

## Publisher’s Note

All claims expressed in this article are solely those of the authors and do not necessarily represent those of their affiliated organizations, or those of the publisher, the editors and the reviewers. Any product that may be evaluated in this article, or claim that may be made by its manufacturer, is not guaranteed or endorsed by the publisher.
