# Supplementary figures and images for: Chicken-Specific Kinome Analysis of Early Host Immune Signaling Pathways in the Cecum of Newly Hatched Chickens Infected With Salmonella enterica Serovar Enteritidis
Source: Front Cell Infect Microbiol. 2022 Jun 30;12:899395. doi: 10.3389/fcimb.2022.899395 (PMC9279939; doi:10.3389/fcimb.2022.899395)

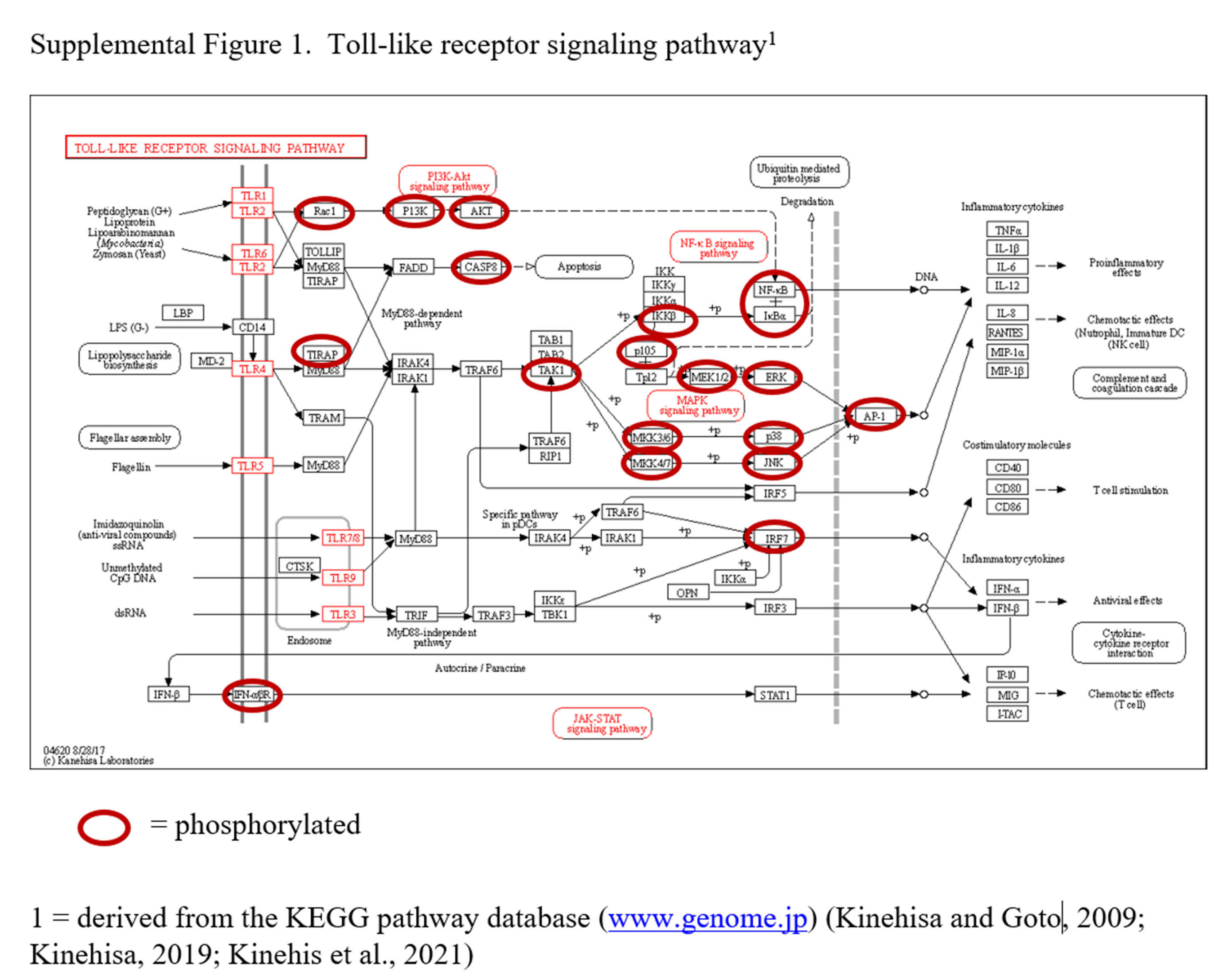

Supplement: Supplementary file 1 [file Image_1.tif]

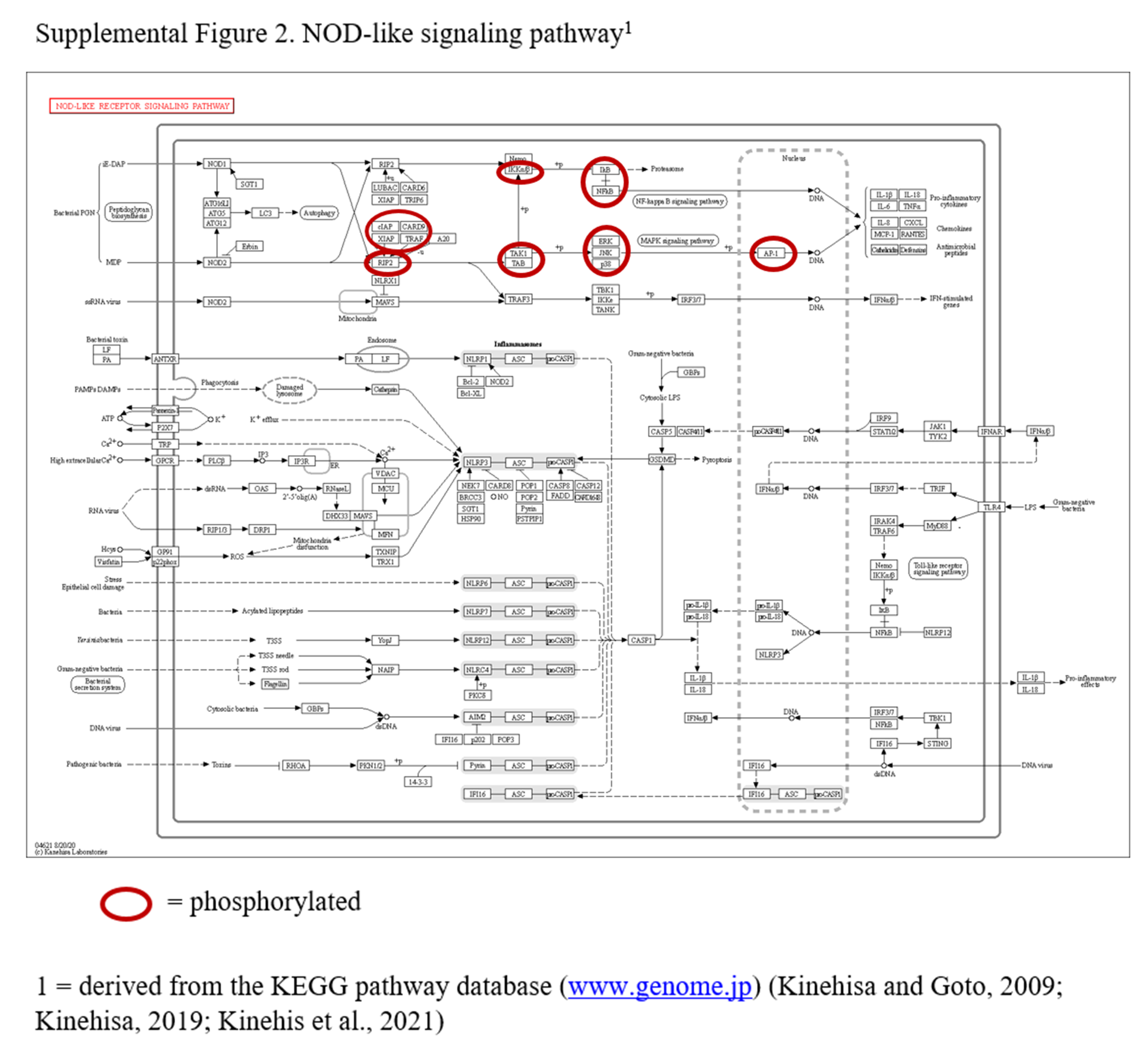

Supplement: Supplementary file 2 [file Image_2.tif]

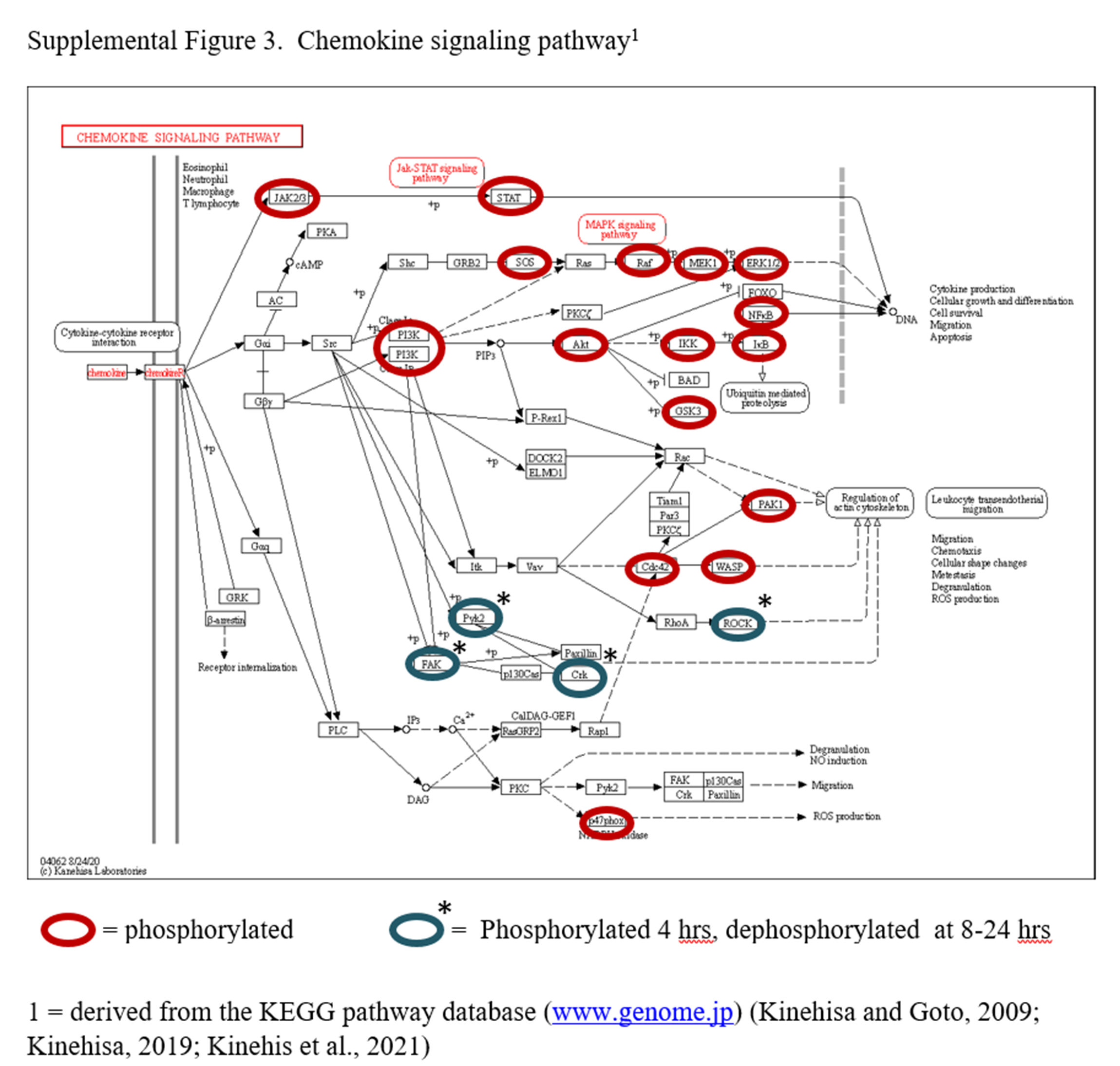

Supplement: Supplementary file 3 [file Image_3.tif]

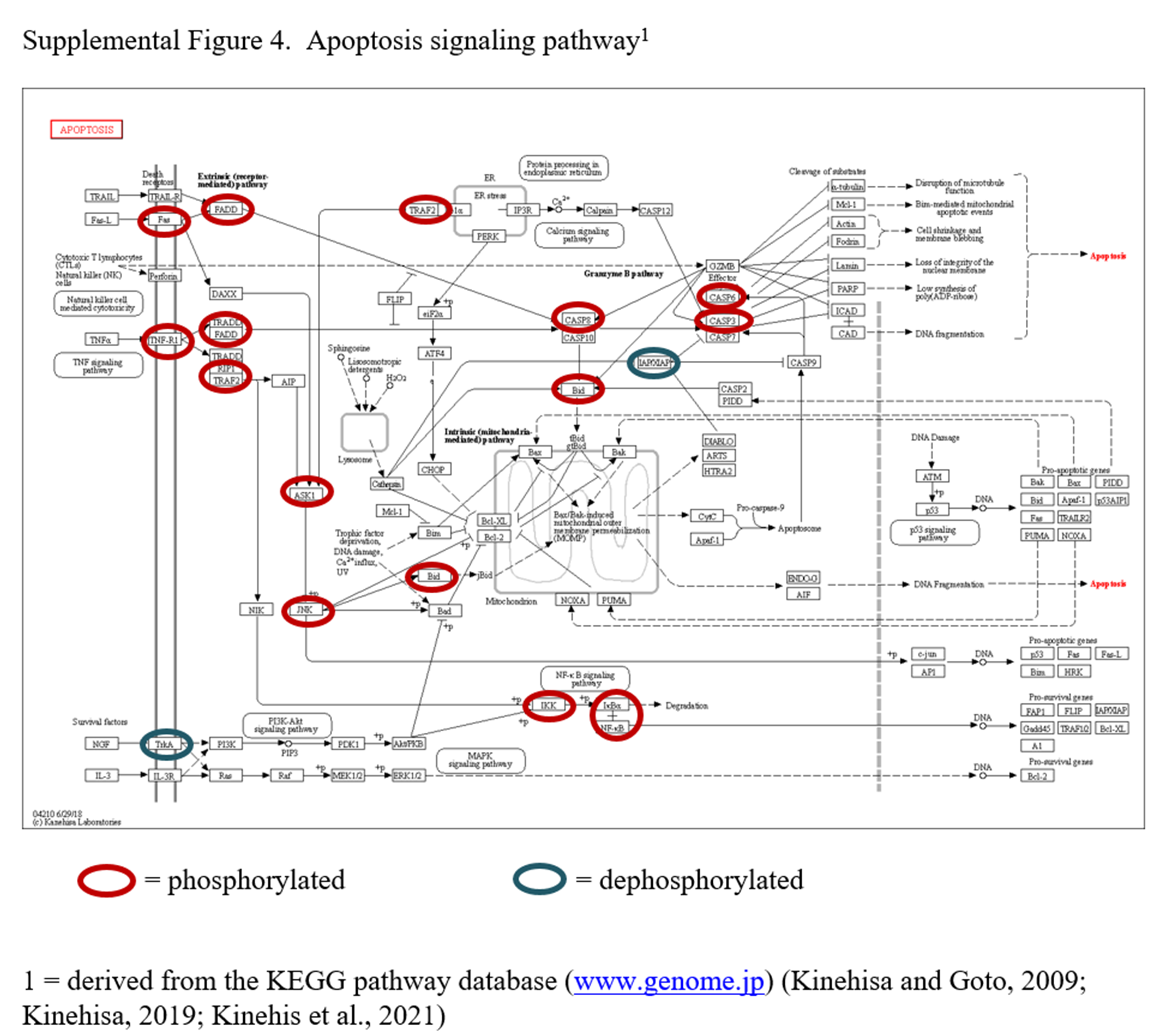

Supplement: Supplementary file 4 [file Image_4.tif]
